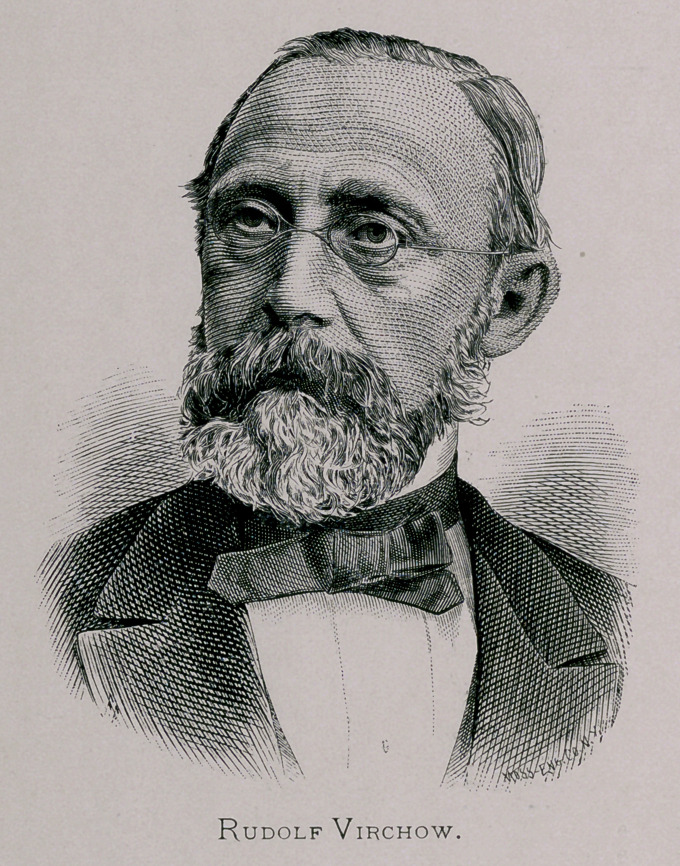# Rudolf Virchow

**Published:** 1885-07

**Authors:** 


### ART. XX.—RUDOLF VIRCHOW.

Virchow was born on the 13th of October, 1821, at Schivelbein, Pommerania, and studied medicine at the University of Berlin, graduating in the year 1843. He at once became Prosector at the Charité. Here began his scientific work, which was at first largely done in connection with his friend Reinhart, with whom he started the world-renowned "Archiv." In 1852 Reinhardt died, since when the "Archiv" has been conducted by Virchow alone, the 100th volume having recently been issued. In the first volumes may be found the work upon which his great reputation mostly depends, for the nuclei of the cellular pathology first appeared as original articles in the JOURNAL, and he who would really understand the true nature of Virchow's work and value his teachings correctly must turn to the Archiv more than to the bound volumes. Here will be found the best criticism of the great French school founded by Bichet, and carried to completion by Laennec, Dupeyton and others, and introduced into Germany by that master of descriptive pathological anatomy, Rokitansky. It is not the purpose of this article to notice all the literary works of Virchow's, but it is to be principally found in the above-mentioned "Archiv," the "Special Pathology, or Encyclopædia," which bears his name as editor, and the three volumes of "Collected Writings," two of which have been recently given to the world. A work which, however, deserves especial mention was a short-lived periodical that bore the name of "Medical Reform," published in connection with Leubuscher in 1848-9. The "Cellular Pathology" has been through numerous editions and published in more languages than any medical book that has ever been written. In connection with it should be mentioned the work upon pathological tumors that still remains, and probably will, uncompleted.

Who shall review the character of Rudolf Virchow? When the history of this century comes to be written and each great man's work critically considered, there will be the names of three men that will tower so far above all others that the lesser and more local lights will be almost lost sight of. These names will be Rudolf Virchow, Charles Darwin and Bismarck. I place them in this order, for it represents to my mind that in which they will rank as benefactors of humanity. Virchow and Darwin have lived for all humanity. Bismarck, for his "liebe Deutschland," but without him German science and human knowledge would not have occupied the advanced position it now does in the world. While Bismarck is the autocrat and often mis-called despot of Germany, he has made of his country the crucible out of which the bonds of the human intellect have found new freedom at the hands of others.

But I am not writing of Bismarck, but of his great political opponent, Virchow. Perhaps my good friend and teacher may object to the comparison, but if Bismarck is the autocrat of Germany, the great pathologist is no less the autocrat in his position and laboratory. Wherever man suffers, wherever death exists, wherever an intelligent medical fraternity treat the ailments of man, Virchow has built a monument as enduring as the intellectual activity of the human race.

Virchow is the humanitarian of German politics. He simply lives beyond his time. Germany is not yet fitted to make practical the ideas of Virchow, but the German has advanced to a better understanding of his manhood through the work of Virchow and the party of progress to which he bebelongs. Without the unity established by Bismarck, the liberty desired by Virchow and others would be much further from future accomplishment than it is at present.

Virchow began his rebellion against existing things very early in his professional career, even before his appointment to a professorship, for in 1849 he was removed from his position by the Ministry "for political reasons,"—a lucky thing for medicine and humanity—and called to Wurzburg, where some of his best work was done.

Now, mark the difference between a republic and the *then despotic*, but still intelligent, monarchy. Virchow had made his name, and the world was beginning to find out that truth had found a new apostle that had stirred the healing waters in the pool of medical stagnation at Wurzburg. If the sick, the lame and the blind did not go there to await the stirring of the waters, those who were to become their medical attendants did, and it is sure that "never man spake" as this new apostle spoke, and never did truths more needed fall upon willing ears, or become fixed in more earnest minds, who, like the apostles of old, went forth from this little German town to spread the glad tidings of *what disease is* over the world. Could such a man be neglected? His politics had not changed; his future has shown us that he was ready to do and dare, do for the good of humanity, whether in facing disease in his daily laboratory work, or studying epidemics wherever sent, or in opposing the government in favor of more liberty in the halls of the German Reichstag, or at the political meetings in Berlin. The masses, those who feel they are down, and want to get up, but do not know how, had as good and true a champion, the government as bitter and obstinate an opponent in 1856 as they have to-day.

Did that have any influence on that government? No! The man best fitted for special work always finds recognition in Germany, no matter what his politics, and does not fear losing his place, no matter how many changes there may be in administrations.

Virchow was called again to Berlin, and received the Professorship of Pathology at the University.

What need have I to say more? And yet a few words may not be out of place by one who knows the master well, and who, even though absent, still sits at his feet.

In person Virchow is of medium height and rather inclined to spareness. In physique, but few men can compare with him in ability to do protracted work; even at his time of life, there are few young men that could hold out and work with him for a succession of days. He is, what one would expect, a simple, straightforward man, and as I have said, an autocrat to those who come in professional connection with him. "Knowledge is power," and it is but doing justice to the man to whom we all owe so much to say that he knows his power and makes you feel it. Still, like every other German, even to Bismarck, of whom very many years in Germany have made it necessary at times to ask favors, Virchow has been to every one who wanted to learn, kindness itself, and even though driven with the almost innumerable calls upon him, I have never seen the time that I was refused, or that I could not obtain all the personal instruction I desired.

Virchow is not eloquent, but logical, profound and clear. He is not only the best analytical pathological anatomist the world has as yet produced, but also the first of the profession that can truly be called a medical philosopher.

Virchow is a skeptic, and hence will be found continally weighing the evidence. He by no means accepts all the new things that have come up, and yet utters no senseless condemnation, as is too often the case with those who have grown old in the ranks. Though his body has aged, his intellect retains all the freshness and vigor of youth.

He is not the person for the ordinary student to study under, or for one to hear who does not thoroughly understand the language, as well as being already up in patho-anatomy. He is too philosophical for such, but to one who is himself a student the master is the teacher *par excellence.*

## Figures and Tables

**Figure f1:**